# Bacterial Heat-Stable Enterotoxins: Translation of Pathogenic Peptides into Novel Targeted Diagnostics and Therapeutics

**DOI:** 10.3390/toxins2082028

**Published:** 2010-08-05

**Authors:** Jieru E. Lin, Michael Valentino, Glen Marszalowicz, Michael S. Magee, Peng Li, Adam E. Snook, Brian A. Stoecker, Chang Chang, Scott A. Waldman

**Affiliations:** 1 Department of Pharmacology and Experimental Therapeutics, Thomas Jefferson University, 132 South 10th Street, 1170 Main, Philadelphia, PA 19107, USA; Email: egerialin@gmail.com (J.E.L.); Michael.Valentino@jefferson.edu (M.V.); Michael.Magee@jefferson.edu (M.S.M.); Peng.Li@jefferson.edu (P.L.); Adam.Snook@jefferson.edu (A.E.S.); steck018@gmail.com (B.A.S.); 2 School of Biomedical Engineering, Science & Health Systems, Drexel University, Philadelphia, PA 19104, USA; Email: gmarsz@gmail.com (G.M.); chang.square@gmail.com (C.C.); 3 Department of Radiation Oncology, University of Pennsylvania, Philadelphia, PA, 19104, USA

**Keywords:** heat-stable enterotoxins (STa), guanylyl cyclase C, guanylin, uroguanylin, colorectal cancer, hormone insufficiency, hormone replacement therapy, tumor vaccine, irritable bowel syndrome, biomarker, targeted delivery

## Abstract

Heat-stable toxins (STs) produced by enterotoxigenic bacteria cause endemic and traveler’s diarrhea by binding to and activating the intestinal receptor guanylyl cyclase C (GC-C). Advances in understanding the biology of GC-C have extended ST from a diarrheagenic peptide to a novel therapeutic agent. Here, we summarize the physiological and pathophysiological role of GC-C in fluid-electrolyte regulation and intestinal crypt-villus homeostasis, as well as describe translational opportunities offered by STs, reflecting the unique characteristics of GC-C, in treating irritable bowel syndrome and chronic constipation, and in preventing and treating colorectal cancer.

## Abbreviations

ABC: Avidin Biotin Complex
CFTR: cystic fibrosis transmembrane conductance regulator
cGMP: Cyclic guanosine monophosphate
CMA: cancer mucosa antigens
CNG: cyclic nucleotide-gated channel
COX-2: cyclooxygenase-2
GC-C: guanylyl cyclase C
IBD: inflammatory bowel disease
IBS: irritable bowel syndrome
MRP: multidrug resistance proteins
PDE: phosphodiesterase
PGE: prostaglandin
PKA: cAMP-dependent protein kinase
PKG: cGMP-dependent protein kinase
PLA2: phospholipase A2
pRb: phosphorylated retinoblastoma
SBM: spontaneous bowel movement
ST: heat-stable enterotoxin

## 1. Introduction

### 1.1. Bacterial heat-stable enterotoxins

Bacterial heat-stable enterotoxins (STs) first came to attention in the 1970s after heat-inactivation of cultures of bacteria isolated from patients suffering from diarrhea failed to eliminate enterotoxigenic activity [1,2]. Two families of heat-stable enterotoxins have been identified: STa (or STI) and STb (or STII), which differ by their physicochemical and biologic characteristics [1,3]. Although STa and STb were both discovered in intestinal bacterial strains isolated from humans, STb is produced by bacterial strains that preferentially inhabit pigs [4]. Further, STa induces diarrhea through a cyclic nucleotide-dependent mechanism, while STb induces secretion through a cyclic nucleotide-independent mechansim [5]. STa, hereon referred to as ST, will be the only heat-stable enterotoxin discussed in this review.

**Figure 1:**
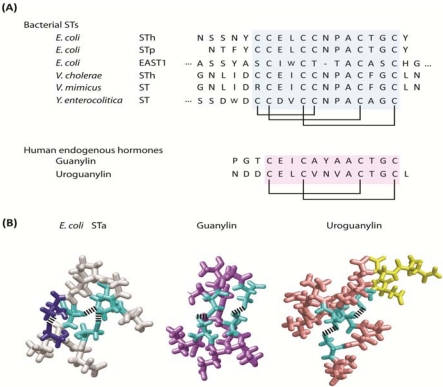
Bacterial heat-stable enterotoxin, guanylin and uroguanylin. (**A**) Primary structures of heat-stable enterotoxins produced by various pathogenic bacteria and human homologs, guanylin and uroguanylin. Conserved core regions are highlighted (blue for STs; pink for hormones) with disulfide bridges indicated; (**B**) Three-dimensional structures of *E. coli* STa (grey), guanylin (purple) and uroguanylin (pink) conserved regions. Non-conserved regions in uroguanylin are labeled in yellow. Cysteines linked by disulfide bonds conserved among three peptides are colored in cyan. Cysteines linked by the extra-disulfide bond, providing ST greater potency than guanylin and uroguanylin, are colored in blue.

STs are produced by a variety of enteric pathogenic organisms, including diarrheagenic *Escherichia coli* (*E. coli*), *Vibrio cholerae*, *Vibrio mimicus*, *Yersinia enterocolitica*, *Citrobacter freundii*, and *Klebsiella pneumoniae* [1,2,6,7,8,9,10]. STs are translated as precursor peptides, which undergo intracellular proteolytic processing to active peptides from 17 to 53 amino acids [11]. Isoforms of ST share a conserved *C*-terminal region of 13 amino acids containing three disulfide bonds responsible for heat stability and biological activity (Figure 1) [11]. Consistent with the proposed function of ST as an essential survival factor facilitating escape of bacteria from nutrient-poor to nutrient-rich environments, synthesis and secretion of ST is reduced in a milieu enriched in glucose while depletion of this sugar stimulates ST production and secretion [12,13]. Investigation of the pathogenesis underlying diarrhea produced by ST ultimately revealed two intestinal paracrine hormones, guanylin and uroguanylin, and the receptor for these homologous peptides, guanylyl cyclase C (GC-C), encoded by the gene *GUCY2C* [14].

### 1.2. Molecular mimicry, convergent evolution and the guanylyl cyclase C paracrine hormone axis

Guanylyl cyclase C (GC-C), the only identified receptor for ST, belongs to the guanylyl cyclase family of receptors that catalyze the conversion of GTP to cGMP upon activation [14]. Guanylyl cyclases are found in two subcellular compartments in mammalian cells: soluble guanylyl cyclases (sGC) are completely intracellular and particulate guanylyl cyclases (pGC), also known as receptor-linked guanylyl cyclase, span the plasma membrane [14]. GC-C is one of the seven isotypes of particulate guanylyl cyclases (GCA to GCG) that exhibit conserved domain structures including an extracellular ligand binding domain, a single transmembrane domain, an intracellular kinase homology domain, and a catalytic domain that produces cGMP [14]. 

GC-C is primarily expressed in intestinal mucosal cells from the duodenum to the rectum [15,16] where it exists as a pre-formed homo -dimer or -trimer [17] located within apical membranes of epithelial cells populating the crypt-villus axis. The two defined endogenous ligands of GC-C, the hormones guanylin and uroguanylin, share significant homology with ST (Figure 1) [18,19]. Similar to ST, the tertiary structure of these peptides is stabilized by intra-chain disulfide bonds, which are essential for biological activity [20,21,22]. Additionally, guanylin and uroguanylin also are synthesized as pro-peptides [23], but unlike ST, these endogenous peptides undergo proteolytic processing following secretion [22,24,25]. These considerations suggest that ST and enterotoxigenic diarrhea are prime examples of molecular mimicry and convergent evolution. Here, bacteria have co-opted a normal mammalian physiologic function, the regulation of intestinal fluid and electrolyte homeostasis, to produce an evolutionary population survival scheme that guarantees the adequacy of nutrient resources and dissemination into new environments and hosts.

### 1.3. Guanylyl cyclase C and enterotoxin signaling circuits

Activation of GC-C stimulates a rise in intracellular cGMP, which binds and activates its three downstream effectors [17]: cGMP-dependent protein kinases (PKGs), phosphodiesterases (PDEs) and cyclic nucleotide-gated (CNG) channels. High intracellular levels of cGMP can also cross-activate cAMP-dependent protein kinases (PKA)[26]. PKGs are the principle intracellular mediators for cGMP signaling [27]. PKG is expressed in nearly all tissues, with highest expression in lung, cerebellum, smooth muscle, platelets, and intestinal mucosa [28,29]. PKG consists of two distinct isoforms: PKG type I and type II. PKG I is located within the cytoplasm of most cell types, whereas PKG II is exclusively expressed within plasma membranes in bone, kidney, brain, and intestine [28,29,30,31]. Within the intestinal epithelium, PKG II displays a rostral-caudal gradient of expression, with highest levels found in small intestine and lowest levels found in the distal colon. Intestinal PKG II expression also displays a crypt-villus gradient with highest expression in the villi and lowest expression in crypts [32]. Two closely related PKG I isoforms (type Iα and Iβ), which arise from alternative splicing of the *N*-terminal region of the PKG I gene have been purified, cloned, and expressed [33,34,35,36,37,38]. Type I and type II PKG isoforms are homodimers of ~75 and 86 kDa monomers, respectively, consisting of (1) an *N*-terminal domain regulating kinase autoinhibition, autophosphorylation and subcellular localization; (2) regulatory domains with allosteric cGMP binding sites and (3) catalytic domains catalyzing the transfer of the γ-phosphoryl group of ATP to various protein substrates [39]. Beyond the primary signal carrying and amplification capacity of PKGs, cGMP signals also are shaped by PDEs, which degrade cGMPs to 5’ GMPs, and multidrug resistance proteins (MRPs), which are ATP-dependent active transporters pumping out cGMP. These enzymes maintain intracellular cGMP concentrations within a narrow physiologic range, essentially serving as key terminators of guanylyl cyclase signaling [40].

## 2. Enterotoxin Signaling, Irritable Bowel Syndrome and Chronic Constipation

### 2.1. Enterotoxins, GC-C and fluid-electrolyte homeostasis

**Figure 2:**
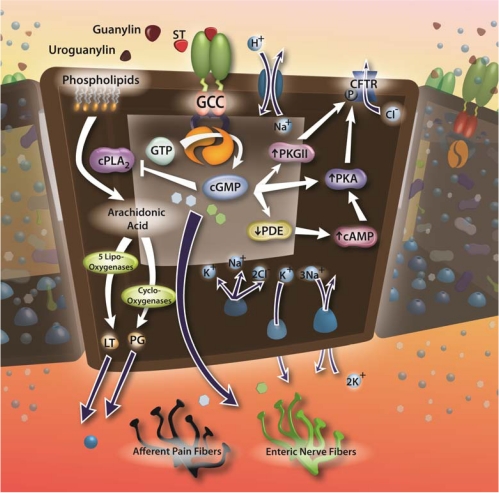
GC-C signaling for fluid/electrolyte secretion, inflammation, gut motility, and nociception. GC-C is activated by binding of bacterial heat-stable enterotoxins (STs) or the endogenous ligands, guanylin and uroguanylin. Ligand binding to the extracellular domain of GC-C activates the intracellular catalytic domain converting GTP to cGMP. Intracellular cGMP accumulation leads to fluid and electrolyte secretion through direct activation of cGMP-dependent protein kinase II (PKG II) and downregulation of phosphodiesterase (PDE)-driven cAMP hydrolysis leading to increased activation of cAMP-dependent protein kinase (PKA). PKG II and PKA phosphorylate the cystic fibrosis transmembrane conductance regulator (CFTR) producing channel opening, leading to an efflux of Cl^−^ ions and secondary efflux of water. Intracellular cGMP accumulation also decreases pro-inflammatory signaling by inhibiting phospholipase A2, decreasing the liberation of the leukotriene (LT) and prostaglandin (PG) precursor, arachidonic acid, from membrane phospholipids. GC-C activation also enhances intestinal myoelectric activity and decreases afferent pain fiber firing, presumably through the release of specific soluble mediators, which stimulate surrounding dendritic nerve endings and alter neuronal firing rates.

Maintenance of fluid and electrolyte homeostasis is critical for cellular biochemical processes and organ function, and the intestine is one of the major organs controlling this balance. Discovery of the role of GC-C in fluid-electrolyte homeostasis is attributed to the observation that ST is a principal cause of enterotoxigenic diarrheal disease in humans and animals worldwide [38,39,40]. Physiologically, guanylin and uroguanylin regulate intestinal fluid and electrolyte homeostasis through cGMP accumulation, which activates PKGII leading to the phosphorylation of the cystic fibrosis transmembrane conductance regulator (CFTR), producing Cl-, HCO3-, and water secretion [14]. ST induces secretory diarrhea by exploiting this physiologic mechanism, and the enhanced affinity of ST for GC-C produces supra-physiologic induction of cGMP accumulation and extensive water and electrolyte secretion (Figure 2) [14,41,42]. Notably, systemic fluid and electrolyte homeostasis is maintained beyond intestine. The natriuretic and diuretic effects induced by uroguanylin and guanylin in the kidney still remain intact in GC-C deficient mice [43,44]. Therefore, GC-C-deficient mice do not exhibit significant systemic fluid-electrolyte imbalance [45]. 

### 2.2. Enterotoxins and irritable bowel syndrome

Besides enhancing gut fluid and electrolyte secretion, infections with enterotoxigenic bacteria, including *V. cholerae* and *E. coli*, alter gut motility [46,47,48], and administration of enterotoxins produced by these bacteria, including STs, stimulate intestinal myoelectric activity [46,49,50]. Indeed, downstream effects of ST signaling are mediated, in part, by stimulation of the enteric nervous system. Mechanical (vagotomy) or neuropharmacological (tetrodotoxin, lidocaine, *etc.*) disturbance of enteric neuronal activation, as well as antagonism of gut acetylcholine, serotonin, prostaglandin, and nitric oxide signaling, significantly reduces ST-induced GI secretion [51,52,53,54,55,56,57]. While the mechanism by which ST signaling stimulates enteric neurons is still unclear, GC-C activation by STs in apical epithelial cells may release specific mediators, which stimulate surrounding nerve endings and alter neuronal firing rates (Figure 2) [58,59].

Due to its ability to induce secretion and enhance gut motility, pharmacological modulation of GC-C signaling has been investigated as a possible treatment paradigm for chronic constipation and constipation-predominant irritable bowel syndrome (IBS-C). Irritable bowel syndrome (IBS) is a functional bowel disorder, which is associated with abdominal pain and altered bowel activity. Unlike inflammatory bowel disease (IBD), IBS is not associated with increased inflammation or other pathologic organic changes within the GI tract [60]. Rather, a diagnosis of IBS is based on specific criteria including recurrent abdominal pain or discomfort, relief with defecation, and change in stool frequency and appearance [61]. Diarrhea- (IBS-D) and constipation-predominant IBS (IBS-C) are the two primary subtypes of IBS, with a mixed subtype (IBS-M) occurring less frequently [60]. 

Constipation and IBS are prevalent in North America, where they have been estimated to affect 12–19% and 5–10% of the population, respectively, with a higher prevalence among females [62,63,64,65,66]. Both conditions are associated with a considerably reduced quality of life [67,68], elevated healthcare costs [69,70,71], and increased disability claims and work absenteeism, which place an economic burden on both patients and employers [72]. The first approach to treating these conditions is diet and lifestyle changes, including increasing exercise and dietary fiber intake [73,74]. Pharmacotherapy with prokinetic and antispasmodic agents are often utilized when lifestyle and dietary changes fail to alleviate symptoms [60]. However, while these agents provide brief symptomatic relief, they do not treat the underlying pathophysiology.

### 2.3. Enterotoxin analogs for chronic constipation

Linaclotide (MD-1100 acetate) is a synthetic 14-amino acid analogue of ST that binds to and activates GC-C and dose-dependently enhances gut secretion and motility in rodent models [75]. Furthermore, linaclotide has anti-nociceptive properties in rodent models of visceral hypersensitivity, indicating that the drug may both improve intestinal motility and discomfort in patients suffering from constipation [76]. In Phase I trials, linaclotide at single oral doses of 30–3000 μg or multiple doses of 30–1000 μg was safe and well-tolerated with no evidence of systemic exposure [77,78]. In the first phase II randomized clinical trial (RCT) of linaclotide utility in the treatment of constipation, 36 women with IBS-C were treated with oral linaclotide (100–1000 μg once daily). At a daily dose of 1000 μg, linaclotide significantly decreased ascending colon emptying half-time (p = 0.015) and enhanced overall colonic transit at 48 hours (p = 0.02), while both doses accelerated the time to first bowel movement (p = 0.013), increased stool frequency (p = 0.037), decreased stool consistency (p < 0.001), and improved ease of stool passage (p < 0.001) without serious adverse effects [79]. In a follow-up study, 42 patients with chronic constipation were randomized to linaclotide (100, 300, or 1,000 μg) or placebo once daily for two weeks. In this study, linaclotide produced a dose-dependent improvement in spontaneous bowel movement (SBM) frequency (p < 0.05), stool consistency scores, and straining scores, in addition to improving abdominal discomfort, severity of constipation, and overall relief with only mild-to-moderate GI side effects, predominantly diarrhea, being reported [80].

In a subsequent large, multi-center trial of 310 patients with chronic constipation treated with linaclotide (75–600 μg daily) for four weeks, all doses improved the weekly rate of spontaneous bowel movements (SBM) and complete spontaneous bowel movements (CSBM) in addition to improving stool consistency, straining, abdominal discomfort, bloating, and quality of life [81]. Finally, in a recent press release, Ironwood Pharmaceuticals, Inc. and Forest Laboratories, Inc announced that, in two phase III trials of >600 patients with chronic constipation, 12 weeks of linaclotide therapy (133–266 μg per day) doubled average weekly CSBMs, tripled average weekly SBMs, and significantly improved bloating, abdominal discomfort, stool consistency, straining, and constipation severity (p < 0.001), with only minor GI side effects [38].

Currently, further trials of linaclotide therapy for the treatment of constipation are scheduled, and new drugs targeting the GC-C signaling axis for the treatment of chronic constipation and IBS-C are in preclinical development. Among these newer agents is SP-304, a synthetic analogue of the endogenous GC-C ligand, uroguanylin, and SP-333, a synthetic GC-C agonist (Synergy Pharmaceuticals Inc.). These agents are currently in preclinical trials for the treatment of GI diseases, including chronic constipation, IBS-C, and inflammatory bowel disease, and oral administration of SP-304 to rodents has been observed to both promote intestinal secretion and ameliorate GI inflammation [82].

### 2.4. Targeting GC-C in chronic inflammation

Nitric oxide has anti-inflammatory effects, inhibiting leukocyte adhesion and oxidant production [83]. In that context, pharmacologic inhibitors of phosphodiesterases, which degrade cGMP, have beneficial effects in mouse models of inflammatory bowel disease [84,85]. Similarly, the uroguanylin analogue, SP-304, produces anti-inflammatory effects, associated with downregulation of pro-inflammatory cytokines including IL-4, IL-5, IL-23, and TNF, in mouse models of ulcerative colitis. Moreover, SP-304 also down-regulated cyclooxygenase-2 (COX-2) production of the inflammatory mediator prostaglandin E2 (PGE2)[82]. The inhibitory function of cGMP on inflammatory prostaglandin production may due to a decrease in intracellular arachidonic acid levels. Arachidonic acid, the precursor for prostaglandin synthesis, is liberated from membrane phospholipids via cleavage by phospholipase A_2_ (PLA_2_). Interestingly, PKG phosphorylates and inhibits PLA_2_ [86]. However, the precise mechanisms underlying the anti-inflammatory activity of SP-304 activation of GC-C remain to be determined (Figure 2).

## 3. Targeting GC-C to Prevent Colorectal Cancer

### 3.1. Dynamics of intestinal epithelial cells and crypt-villus homeostasis

The intestinal epithelium lining the gastrointestinal tract is characterized by coordinated homeostatic programs comprising a developmental continuum integrating proliferation, differentiation, metabolic maturation and apoptosis along the crypt-villus axis. Crypts harbor stem cells at their base, which continuously regenerate progenitor cells destined to differentiate along secretory (goblet, Paneth, and enteroendocrine cells) or absorptive (enterocytes) lineages. This transition from proliferation to lineage commitment is associated with metabolic reprogramming from glycolysis to oxidative phosphorylation as the cells migrate toward the surface [87]. Metabolic reprogramming reflects the energy demands for different compartments, subserving the specific balance between proliferation and differentiation. In the crypt, cell division requires rapidly available energy supplies from glycolysis, while mitochondrially-mediated metabolism exploits the efficiency of ATP production by oxidative phosphorylation, supporting catabolic demands in mature cells in the differentiated compartment [88,89]. Following this transition, goblet, enteroendocrine cells and enterocytes continue to migrate to the tips of the villi and ultimately undergo apoptosis or anoikis [90,91,92]. In contrast, Paneth cells migrate down to the base of the crypt where they reside. The distinctive regenerative characteristic of the intestinal epithelium establishes a vertical axis representing a life cycle continuum, from cell birth to death. Dysregulation of this homeostatic process is intimately linked with intestinal tumorigenesis [89].

Enterocytes, comprising the majority of the intestinal epithelial monolayer, develop well-organized microvilli brush borders, containing key functional proteins mediating cognate digestive and absorptive functions [91]. Interspersed among enterocytes are the hormone-producing enteroendocrine cells, which comprise the largest endocrine system in the body in terms of both cell quantity and variety of hormones produced. Although enteroendocrine cells share biochemical similarities with neurons, they are derived from the same progenitor cells as other epithelial cells, originating from the endoderm. These cells secrete hormones supporting intestinal neuromuscular function, digestion, secretion, and central regulation of food intake as well as other systemic processes [93,94,95,96,97,98]. Goblet cells secrete mucin, which forms a mucus layer protecting intestinal surfaces and facilitating nutrient digestion and absorption by enterocytes [97]. Paneth cells, located only in small intestine, secrete antimicrobial peptides and growth factors into the lumen [99], forming a physical and functional barrier defending against bacterial invasion and intestinal tumorigenesis through innate immune responses [99]. 

### 3.2. Enterotoxigenic signaling pathways and intestinal tumorigenesis

**Figure 3:**
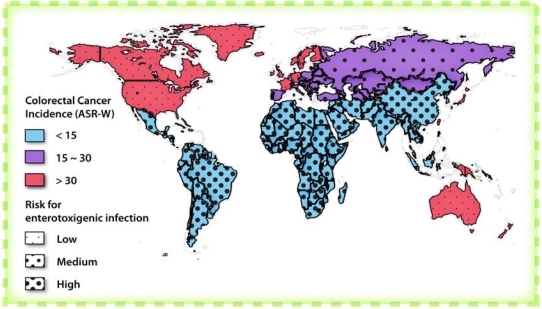
World map comparing the prevalence of enterotoxigenic bacterial infections and colorectal cancer. This map highlights the inverse relationship between the prevalence of enterotoxigenic bacterial infections and incidence of colorectal cancer worldwide. The prevalence of enterotoxigenic infections is represented by the intensity of dots while the incidence of colorectal cancer is represented by color. Adapted from [97].

**Figure 4:**
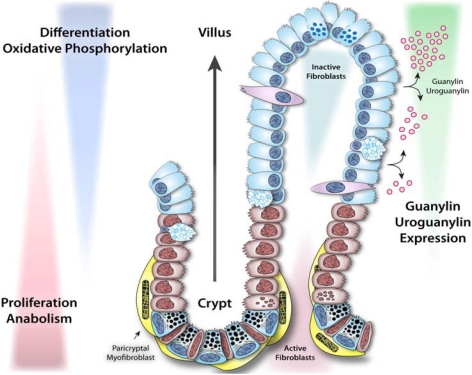
GC-C signaling regulates homeostatic programs along the crypt-villus axis in the intestinal epithelium. The intestinal epithelium undergoes continuous regeneration and differentiation along the crypt-villus axis. Stem cells residing near the bottom of crypts give rise to rapidly proliferating progenitor cells, which subsequently differentiate into functional enterocytes. Guanylin and uroguanylin are secreted in a gradient along the crypt-villus axis, while guanylin is primarily secreted in colon and uroguanylin is mainly secreted in small intestine. Ligand activation of GC-C signaling reprograms proliferative and metabolic circuits in intestinal epithelial cells and ensures a homeostatic balance between proliferation and differentiation. Adapted from [84].

There is an important functional relationship between circuits mediating enterotoxigenic signaling, intestinal homeostasis, and colorectal tumorigenesis that reflects co-option of critical physiologic cell pathways by bacteria. There is an under-appreciated inverse epidemiologic relationship between the prevalence of enterotoxigenic infections and colorectal cancer worldwide, and geographic regions that have the highest chronic colonization rates have the lowest rates of intestinal tumorigenesis (Figure 3) [100,101]. Also, guanylin and uroguanylin exhibit a pattern of expression along the crypt-villus axis that is associated with the transition from proliferating to differentiated compartments. These gene products are absent in the bottom of crypts (except Paneth cells in the small intestine) but present in the villus compartment in the small intestine and colonic surface epithelial cells (the mature enterocytes) (Figure 4) [14,59,102,103,104,105]. Additionally, guanylin and uroguanylin are the most commonly lost gene products in colorectal cancer, and their loss occurs early along the continuum of transformation [106,107,108,109]. Moreover, elimination of GC-C signaling increases the susceptibility of mice to intestinal tumorigenesis induced by carcinogens or inherited germline mutations [89,110] while, conversely, supplementation with uroguanylin decreases intestinal tumorigenesis in mouse models [101]. 

The effects of GC-C on normal crypt-villus dynamics and corruption of these mechanisms in tumorigenesis reflect a central role for cGMP and downstream effectors in coordinating intestinal epithelial cell homeostasis. GC-C activation inhibits proliferation of intestinal cells by prolonging the cell cycle through a cGMP-dependent mechanism. Hyperproliferation and acceleration of the epithelial cell cycle in GC-C deficient mice is associated with an increase in mediators promoting the G1/S transition (cyclin D1, pRb) and a decrease in cell cycle suppressors (p27), accompanied by hyperplasia of the crypt compartment, reflecting an increase in the number of progenitor cells. Also, mice deficient in GC-C signaling demonstrate an expansion of the proliferating crypt compartment [89] associated with a defect in differentiation with preferential commitment along the enterocytic, compared to the secretory, lineage [111]. Selective impairment in maturation of the secretory lineage reflects a role for GC-C in regulating intestinal cell differentiation by discrete molecular mechanisms, including interaction with transcription factors specifying secretory lineage commitment including Hes-1 and Math [111,112]. Elimination of GC-C expression also induces genomic instability in intestinal epithelial cells, increasing DNA double strand breaks, loss of heterozygosity, and point mutations in genes central to tumorigenesis, including APC and β-catenin [110]. Beyond accelerating the cell cycle and corrupting DNA damage sensing and repair, loss of GC-C signaling reprograms metabolism to the Warburg phenotype, directing ATP generation through aerobic glycolysis associated with a reduction in mitochondria and increases in reactive oxygen species which directly damage DNA [87,89,113].

In the context of the established role of accumulated genetic alterations reinforcing genomic instability in carcinogenesis, loss of GC-C ligands early in tumorigenesis underscores the mechanistic contribution of dysregulated GC-C signaling in colorectal cancer. Mechanisms by which GC-C contributes to genomic integrity, including damage protection, detection and assessment, mutation repair, and the associated coordination of replicative decision-making are currently being explored. However, proliferative restriction and genomic quality control reflect reinforcing mechanisms by which GC-C opposes tumorigenesis [110]. Indeed, accelerated progression through G1 phase and premature entry into S phase are necessary for heritability and amplification of genetic instability [113,114]. Together, these observations suggest that GC-C is a lineage-specific tumor suppressor normally involved in the spatiotemporal patterning of the intestinal crypt-villus axis whose silencing, reflecting loss of expression of paracrine hormones, corrupts downstream processes universally underlying neoplastic transformation [87,89,100,110,115,116,117] Accordingly, colorectal tumorigenesis regulated by GC-C signaling suggests a novel pathophysiological paradigm in which colorectal cancer initiates, in part, as a disease of paracrine hormone insufficiency.

### 3.3. GC-C paracrine hormone replacement to prevent colorectal cancer

The novel roles of GC-C signaling in maintaining intestinal proliferative and metabolic homeostasis and suppressing intestinal tumorigenesis combined with the loss of GC-C ligands as an early event in intestinal neoplasia underscores a novel therapeutic paradigm for targeted colon cancer prevention and treatment through oral supplementation of GC-C ligands. Activation of the dormant tumor-suppressing receptor is anticipated to coordinately rescue cell cycle restriction and reprogram the Warburg glycolytic metabolic phenotype to inhibit intestinal tumorigenesis. Indeed, activation of GC-C signaling in human colon cancer cells inhibits cell proliferation by transient arrest of cell cycle and DNA synthesis, quantified by cell growth, colony formation, and ^3^H-thymidine incorporation [100,111,117,118]. GC-C signaling also induces a G1-S transition delay, quantified by flow cytometry and BrdU incorporation. This delay in cell cycle progression occurs in the absence of apoptosis, measured by TUNEL analysis and DNA laddering, or necrosis, determined by trypan blue exclusion and lactate dehydrogenase release. Cytostasis induced by GC-C ligands is specifically mediated by the accumulation of cGMP, as it is mimicked by the cell-permeant analog 8-Br-cGMP and reproduced and potentiated by the cGMP-specific phosphodiesterase inhibitor zaprinast, but not the inactive ST analog TJU1-103 [100,101,102,103,104,105,106,107,108,109,110,111,117]. Cytostasis induced by GC-C signaling is associated with altered expression of cell cycle mediators including cyclin D, pRb, and p27 regulating the transition through G1-S phase [89,100,111,117]. 

Activation of GC-C signaling also reverts the tumorigenic Warburg metabolic phenotype in human and murine colon cancer cells. GC-C signaling inhibits glycolysis and fatty acid synthesis by reducing rate-limiting enzymes including glucose transporter 1, hexokinase, pyruvate kinase, acetyl-CoA carboxylase and acid citrate lyase, associated with a decrease in glucose uptake and lactate production. Further, activation of GC-C signaling in human colon cancer cells induces expression of critical transcription factors required for mitochondrial biogenesis, including PGC1α, mtTFA, and NRF1 [89]. Moreover, GC-C signaling promotes mitochondrial biogenesis by increasing mitochondrial content, associated with enhanced mitochondrial oxygen consumption, dehydrogenase activity and ATP production [89]. Reversion of the tumorigenic phenotype by GC-C is generalizable, reproduced in numerous human and mouse colon cancer cell lines [89]. Of significance, activation of GC-C signaling suppresses ROS (reactive oxygen species) production, reflecting increased function of the electron transport chain [119,120] or increased production of ROS scavengers [121,122], promoting genetic stability [89].

Interestingly, a study [101] using *Apc^Min/+^* mice suggests that supplementation of an endogenous GC-C hormone, uroguanylin, in food and drinking water significantly suppresses intestinal tumorigenesis, including tumor multiplicity and tumor size in both small intestine and colon [101]. Of significance, this study suggests that induction of GC-C signaling prevents tumor initiation and progression by promoting apoptosis, rather than restricting cell proliferation. In that context, it is noteworthy that GC-C signaling prevents, rather than induces apoptosis in human colon cancer cells [123]. Thus, mechanisms underlying inhibition of tumorigenesis by uroguanylin administration remain unresolved [101,116,117].

In summary, these observations suggest that reconstitution of GC-C signaling in human colorectal cancer cells by replacing GC-C ligands inhibits cell proliferation through cGMP-dependent mechanisms by coordinated regulation of the cell cycle, metabolic circuits, chromosomal instability and/or cell death. In the context of universal hormone loss early in colorectal neoplasia, reconstitution of dormant receptor signaling by oral ligand supplementation may prevent initiation and progression of colon cancer [101] by opposing proliferation, metabolic reprogramming, and genomic instability [89,110].

## 4. Diagnostics and Therapeutics Targeted to GC-C for Metastatic Colorectal Cancer

### 4.1. GC-C as a marker of metastatic colorectal cancer

Constitutive expression of GC-C mRNA is universally retained throughout the transformational continuum in intestine, from adenoma to metastatic carcinoma [124]. Primary and metastatic tumors universally retain ST binding and cGMP production, in contrast to extra-intestinal tissues and tumors which do not express GC-C [15,125]. Further, GC-C expression in metastatic colorectal tumors was quantitatively similar to that in primary tumors, but in excess of normal colonic mucosa, quantified by immunohistochemistry [15,125]. Moreover, GC-C mRNA was identified in all primary and metastatic colorectal tumors, but not in extra-gastrointestinal tumors examined to date [124]. Universal over-expression of GC-C, at transcriptional and translational levels, by all primary and metastatic tumors arising from the colon and rectum, but not by extra-intestinal tumors, suggest that GC-C may be a unique biomarker for identifying and targeting metastatic colorectal cancer cells. Similarly, Gold and Freedman demonstrated in their seminal paper [126] that increased carcinoembryonic antigen (CEA) can be utilized as a biomarker for primary colon tumors. This elevated expression of CEA facilitates targeting with CEA-specific antibodies to colorectal tumors [127]. However, CEA is detectable in serum, while GC-C is normally absent in the circulation. Further, circulating CEA levels have been found in cigarette smokers, in patients with benign neoplasms, and in 15–20% of subjects with inflammatory disorders such as ulcerative colitis, Crohn's disease, pancreatitis, liver disease, and pulmonary infections. Such non-specificity renders CEA a less useful biomarker [128].

### 4.2. GC-C qRT-PCR as a molecular marker to stage patients with colorectal cancer

The presence of tumor cells in regional lymph nodes is the single most important prognostic marker of survival in colorectal cancer patients [129,130]. Despite this established relationship, standard histopathologic lymph node screening remains imperfect and patients with node-negative (pN0, stage I and II) disease exhibit five year recurrence rates of ~25%, suggesting the presence of occult metastases in some patients [131]. Also, metastases in lymph nodes represent the principle predictive marker for identifying patients who benefit from adjuvant chemotherapy [132,133,134,135,136]. However, while treatment of stage III patients has been associated with improved survival, its utility in the pN0 population remains uncertain [131,132,133,134,135,136,137,138,139]. These observations emphasize the need for a more accurate assessment of occult metastases in the regional lymph nodes of pN0 colorectal cancer patients to improve current prognostic and therapeutic outcomes.

The expression of GC-C is normally restricted to intestinal epithelia cells. However, its universal over-expression by colorectal cancer cells creates a unique opportunity to utilize the receptor as a biomarker for metastases [15,140,141]. Previous retrospective analyses demonstrated GC-C expression by quantitative reverse transcriptase-polymerase chain reaction (qRT-PCR) and its association with disease recurrence in pN0 patients [142]. These studies formed the basis for a recent trial prospectively exploring molecular analysis of GC-C by qRT-PCR in pN0 colorectal cancer patients to identify occult metastases and define prognostic risk [142]. Indeed, GC-C expression was detected in 87% of pN0 patients, while 13% remained free of tumor cells. Further, 21% of GC-C-positive patients, but only 6% of GC-C-negative patients, developed recurrent disease. Indeed, patients who were GC-C-positive experienced a shorter time to recurrence and a decrease in disease-free survival compared to GC-C-negative patients. Moreover, GC-C qRT-PCR was the most powerful independent prognostic marker of risk for pN0 patients. This study suggests that molecular detection of occult metastases in lymph nodes from pN0 colorectal cancer patients employing GC-C qRT-PCR may improve staging precision and represent an important advancement for the prognostic and predictive management of patients with colorectal cancer. Indeed, the use of this technique in conjunction with established clinical staging modalities will enhance the treatment of patients by better defining individual risk and predicting the benefits of adjuvant chemotherapy.

### 4.3. GC-C to deliver targeted diagnostics and therapeutics to metastatic colorectal cancer

While primary tumors are effectively treated with surgical resection, only 39% of colorectal cancer cases are diagnosed prior to the cancer spreading from the primary site. Early stage patients enjoy a 69% rate of survival over five years, while the five-year rate of survival for cases with distant metastasis upon diagnosis drops precipitously to 11.3% [143]. This direct relationship between occult metastases, which are present but undetected, at the time of diagnosis, and mortality highlights the unmet clinical need for effective imaging of colorectal cancer metastasis to improve the precision of diagnostic staging and identify patients who could derive benefit from adjuvant chemotherapy. It also underscores an essential future goal of developing targeted therapeutic agents that can eradicate metastatic cancer cells without impacting surrounding normal cells, an objective not yet achieved for colorectal cancer. In the context of diagnostic imaging, the current standard utilizes positron emission tomography (PET) to visualize enhanced glycolysis characterizing the Warburg metabolic phenotype in cancer. In that paradigm, 2-[^18^F]fluoro-2-deoxy-D-glucose (FDG) is taken up by tumors at a higher rate than surrounding normal tissues. While this differential uptake supports PET imaging, limited sensitivity and specificity reflect the dependency of all cells on glucose metabolism.

Advanced technology platforms for imaging seek targeted receptor-based molecular probes, rather than metabolic detectors, to improve the sensitivity and specificity of metastatic tumor detection [144,145]. In the context, ST conjugated to a radionuclide retains full potency for GC-C binding, and differential accumulation in subcutaneous and hepatic human colon tumors expressing GC-C, but not in other tissues or tumors that do not express GC-C, can be visualized by gamma camera scintigraphy with extremely high sensitivity and specificity [146,147,148,149,150,151,152,153]. Advantages conferred by specific expression of GC-C only in metastatic tumors in extra-intestinal sites is remarkably enhanced by endocytosis of this receptor following ligand binding [146,153]. Beyond rapid internalization, endocytosis of GC-C deposits cognate ligands and their payloads in the intracellular compartment, after which these receptors recycle back to the surface [146,153]. Thus, continuous exposure to ligand conjugates, for example in the circulation, permits extended accumulation of their payloads intracellularly in tumors, magnifying highly selective diagnostic and therapeutic exposures beyond that achieved by cell surface binding alone [146,153]. Moreover, amplification provided by intracellular accumulation can be further enhanced by employing polyvalent targeting agents, for example, GC-C-directed antibodies modified to carry multiple diagnostic and/or therapeutic agents. In the context of the universal expression of GC-C in colorectal tumors [124,140,142,154], these observations underscore the future utility of GC-C-directed ligands for both detecting and treating metastatic disease.

### 4.4. GC-C-targeted colorectal cancer vaccines

Inducing active immune responses against tumors through vaccination has emerged as an attractive adjuvant approach for secondary prevention of cancer. One major challenge in developing cancer vaccines has been the identification of suitable tumor antigens that can be recognized by the host immune system. Ideally, target antigens would be entirely tumor-specific, mediating recognition and elimination by the immune system without collateral autoimmunity in normal tissues. However, tumors arise from normal tissues and, consequently, express self antigens subject to mechanisms of tolerance which prevent both autoimmunity and antitumor immunity [155].

Thus, tumor vaccine strategies have targeted self-proteins employing a variety of strategies. Oncogenic transformation results from mutation of self proteins, which may provide an immunologically unique (“foreign”) tumor target [156]. Also, oncofetal antigens, which are only expressed during fetal development, may be ectopically expressed after tumor transformation [157]. Similarly, some tumors ectopically express cancer testis antigens, which are normally found only in immune privileged sites, limiting tolerance to these antigens. However, expression of these antigens is rare in epithelial tumors, requiring screening and production of individualized cancer vaccines. Alternatively over-expressed self-antigens including Her2/neu, Mucin-1, EpCAM, and CEA have been targeted in cancer vaccination strategies [158,159,160]. Indeed, a modest clinical impact has been achieved by targeting CEA-expressing colorectal tumors in conjunction with immunostimulatory adjuvants. However, the requirement for adjuvants reveals suboptimal responses to antigen alone, which may reflect central tolerance to self antigens [161]. In fact, expression of many tumor-associated self antigens can be widespread in normal tissues, substantially hindering immune responses to them. Further, expression of these antigens in normal tissues also may enhance immune related toxicities produced by these vaccines. For example, 10% of patients immunized with a multi-peptide melanoma vaccine developed vitiligo [162].

In contrast to conventional antigens, a recently established class of tumor targets, cancer mucosal antigens (CMAs), may be ideal for directed treatment of metastatic cancer. CMAs are expressed exclusively in mucosal tissues, such as the intestinal epithelium, and in tumors derived therein, such as colorectal cancer. Importantly, separation of mucosal and systemic immune responses, reflecting physical and functional barriers, limits immunological cross-talk between compartments [163,164]. Indeed, lymphocyte activation imprints immune cells with chemokine receptor and adhesion molecule expression that retain them in the compartment of activation. For example, T cells activated in skin-draining lymph nodes by Langerhans cells up-regulate CCR4 and E-selectin ligand expression resulting in homing to inflamed skin [165]. In that regard, potential deficits in systemic tolerance toward CMAs, based on their confinement to the mucosal compartment, permit generation of CMA-specific immune responses following systemic immunization. These immune responses may augment anti-tumor immunity with limited autoimmunity due to the inability of systemically activated cells to traffic to the mucosal compartment [87,166,167].

GC-C is the first identified CMA and its normal restriction to the mucosal compartment might permit the generation of GC-C-specific systemic immunity due to attenuated systemic tolerance. Moreover, the universal expression of GC-C in tumors arising from the colon and rectum [124,142,154] may make it an ideal target for effective immunotherapy in all patients. Indeed, preclinical mouse models have demonstrated that mice generate anti-GC-C immune responses when primed with a recombinant adenoviral vaccine expressing the extracellular domain of GC-C. Prophylactic immunization with adenovirus expressing GC-C inhibited tumor growth and prolonged survival in parenchymal lung and liver metastases models [168,169]. Analysis of vaccine-induced immune responses revealed lineage-specific systemic tolerance to GC-C. Immunization induced GC-C-specific CD8^+^ T cell, but not GC-C-specific CD4^+^ T cell or antibody responses, in wild-type mice. In contrast, immunization of mice in which GC-C expression was eliminated (GC-C^−/−^) induced GC-C-specific CD8^+^ T cell, CD4^+^ T cell, and antibody responses. Induction of all arms of the adaptive immune system in GC-C^−/−^ mice indicates an incomplete systemic tolerance in wild type animals, in which only GC-C specific CD8^+^ T cells circumvent toleragenic mechanisms [169]. In contrast, GC-C-specific CD4^+^ T cells are targets of systemic tolerance in wild-type mice and may be deleted during thymic development, differentiate into immunosuppressive regulatory T cells, or rendered anergic in the periphery. 

The generation of GC-C-specific immunity in mice did not produce serum anti-nuclear antibodies or enhancement of immune infiltrates into gastrointestinal tissues [169]. Moreover, maximal GC-C specific CD8^+^ T cell responses generated through heterologous prime-boost vaccination did not exacerbate autoimmunity in a chemically-induced mouse model of inflammatory bowel disease or augment tumorigenesis in colitis-associated or genetic models of colorectal cancer [168]. These observations reinforce the hypothesis that GC-C-specific immunization can promote anti-tumor efficacy without autoimmunity [168]. The absence of autoimmunity likely reflects immune compartmentalization, which limits trafficking of systemically activated immune cells to the mucosal compartment. Systemic immunization producing effective immune responses in the systemic compartment fails to produce mucosal responses protecting the gastrointestinal tract from GC-C-specific autoimmunity [170]. Additional studies characterizing the mechanisms of tolerance in wild type facilitate the development of next-generation vaccines that optimally activate all branches of the adaptive immune response in order to achieve maximal anti-tumor immunity targeted to cancer mucosal antigens.

## 5. Conclusions

The ability of toxins to co-opt normal physiological pathways to produce pathophysiological consequences can be exploited to explore the fundamentals of cell biology and mechanisms of disease. Thirty years after identification of bacterial heat-stable enterotoxins, their study has revealed the novel receptor GC-C and its two endogenous paracrine hormones, guanylin and uroguanylin, and decoded signaling pathways regulating intestinal homeostasis. These studies have identified novel therapeutic and preventive strategies for colorectal cancer, chronic constipation, and inflammatory bowel disease. Furthermore, universal retention of GC-C expression in colorectal tumors offers a unique target for diagnosis, therapy, and vaccine development. The emerging understanding of heat-stable enterotoxins, beyond their induction of diarrheal disease, uniquely exemplifies success in translational research across the continuum from the laboratory bench, to the bedside and beyond to patient populations.
